# Compressive Strength Prediction of Green Concrete with Recycled Glass-Fiber-Reinforced Polymers Using a Machine Learning Approach

**DOI:** 10.3390/polym17202731

**Published:** 2025-10-11

**Authors:** Pouyan Fakharian, Reza Bazrgary, Ali Ghorbani, Davoud Tavakoli, Younes Nouri

**Affiliations:** 1 Institute of Research and Development, Duy Tan University, Da Nang 550000, Vietnam; pouyanfakharian@duytan.edu.vn; 2 School of Engineering & Technology, Duy Tan University, Da Nang 550000, Vietnam; 3 Department of Civil and Environmental Engineering, University of Houston, Houston, TX 77204, USA; 4 Department of Engineering, Payame Noor University, Tehran 193954697, Iran; 5 Materials and Constructions, Department of Civil Engineering, KU Leuven, 3001 Leuven, Belgium; 6 Department of Civil Engineering, Faculty of Engineering, Ferdowsi University of Mashhad, Mashhad 9177948974, Iran

**Keywords:** GFRP, waste, recycled polymer, concrete, reuse, machine learning, XGBoost

## Abstract

Fiber-reinforced polymer (FRP) materials are increasingly used in the construction and transportation industries, generating growing volumes of waste. This study applied a machine learning model to predict the compressive strength of eco-friendly concrete incorporating recycled glass-fiber-reinforced polymer (GFRP) waste. Based on 119 laboratory mixes, the model achieved a good prediction accuracy (R^2^ = 0.8284 on the test set). The analysis indicated that compressive strength tends to decrease at higher GFRP dosages, with relatively favorable performance observed at low contents. The two most influential factors were the water-to-cement ratio and the total GFRP content. The physical form of the recycled material was also important: powders and fibers generally showed positive effects, while coarse aggregate replacement was less effective. This machine learning-based approach offers preliminary quantitative guidance on mix design with GFRP waste and highlights opportunities for reusing industrial by-products in more sustainable concretes.

## 1. Introduction

The reuse of GFRP waste in concrete and pavements provides a route to divert composites from landfill and to reduce the environmental burden of disposal by substituting low-value waste for virgin fillers or aggregates in construction mixes. Mechanical incorporation (shredding or size grading) into pervious concrete, asphalt mastics, or aggregate and filler has been demonstrated as a low-energy, practicable way of keeping most environmental burdens tied to cement production rather than to GFRP processing and transport and allowing recycling of substantial tonnages in pavement applications [1,2,3]. Mechanical recycling is the dominant and scalable route for GFRPs, where comminution and screening are industrially mature for glass-fiber composites and already used at scale [2,3]. Reusing GFRP waste in construction can create economic value, avoiding costly disposal; reduce demand for mined aggregates; and enable end-markets for blade, manufacturing and installation wastes. The approach supports circular economy goals by converting difficult-to-treat thermoset waste into useful construction inputs and, in some product formats, improves product performance and marketability [1,2]. Also, FRP materials have been used in the construction of reinforced concrete buildings due to their resistance to severe environmental exposure. Degradation processes in these materials could cause fiber erosion, matrix cracking, and fiber–matrix debonding, primarily in the peripheral region of FRP rebar. However, under these conditions, the use of FRPs in concrete structures as reinforcements has many advantages [4]. The addition of glass fibers to concrete significantly enhances its dynamic strength properties, including tensile strength, compressive strength, and impact resistance. Studies have shown that increasing the fiber content in GFRC improves its overall strength and toughness under dynamic loads [5,6]. Small replacements of fine aggregate by ground GFRPs or the inclusion of recycled discrete fibers often produce modest strength gains or maintain strength, whereas high-volume aggregate replacement tends to reduce compressive strength. Specific experimental findings show that low-volume substitutions or discrete recycled macro-fibers can improve tensile/flexural metrics substantially, while full replacement of coarse aggregate reduces moduli and peak strength [7,8,9,10]. Macro-fiber reinforcement (using recycled macro-fibers derived from waste GFRPs) increased the flexural strength and toughness of recycled-aggregate concrete: the flexural strength rose from 3.13 MPa to 5.97 MPa at a 1.5% fiber content and the toughness increased from 1.4 J to 77.6 J in one study [7]. Grain-sized GFRP powders used to replace sand showed an optimum low replacement (5% by weight) that improved workability and compressive strength, while higher replacement ratios reduced compressive strength but increased splitting tensile strength in some mixes [9]. Similar optimization-oriented studies have also demonstrated that statistical design methods like the Taguchi approach can effectively identify mix parameters for enhancing strength performance [11]. Recycled GFRPs used as coarse aggregate and graded recycled GFRP aggregate led to decreased compressive strength and elastic moduli with increasing replacement ratios, but reduced post-peak brittleness and altered failure modes compared to natural-aggregate concrete [8]. Fine GFRP powder had little effect on compressive strength, whereas short fiber clusters (longer fibers/higher fiber content) provided modest gains in compressive and splitting tensile strength and improved failure modes [10]. Low replacement rates of recycled materials as discrete fibers should be utilized for structural benefits (toughness and post-crack behavior) rather than full aggregate replacement if the target is to maintain compressive strength [7,8,9,10]. Particle/fiber size and grading should be controlled to match aggregate/mixture design, since grading affects packing, the interfacial transition zone (ITZ), and early-age bonding [8,12].

Zhou et al. reported a maximum flexural strength of 9.83 MPa for mortars reinforced with elaborately recycled GFRP fibers [13]. Baturkin et al. observed flexural strength increases of up to 22% (for 1.75 vol.% fibers after wood removal) and four-times flexural toughness increases for 1.75 vol.% fibers without wood content; the tests were performed on 28-day cured specimens [14]. Revilla-Cuesta et al. reported a flexural strength near 6.1 MPa for mixes containing 1.5% and 6.0% Selectively Crushed Wind Turbine Blade (SCWTB) [15]. El Bitouri et al. found that flexural strength was less affected than compressive strength when sand was replaced by GFRPs at 10% replacement: flexural strength decreased by 37%, while compressive strength decreased by 54%; flexural toughness rose from 0.351 N·m to 0.642 N·m at 15% GFRP replacement [16]. Revilla-Cuesta et al. reported that the splitting tensile strength remained at 4.7 MPa with up to 3.0% SCWTB additions [15]. Hilles and Ziara (who examined AR-GFRPs in high-strength concrete) recorded a splitting tensile increase from 3.06 to 4.92 MPa as the fiber percentage rose from 0.0 to 1.2%, and the flexural strength rose from 4.84 to 7.27 MPa; the tests were performed in accordance with the ASTM standards for compressive, splitting tensile, and flexural strengths [17]. Mixed waste glass powder and glass fiber studies showed splitting tensile and flexural strength increases of 10.7% and 13.5% with 1% GF (glass fiber) addition and further gains when this was combined with 10% WGP (splitting strength: 24.6%, flexural strength: 21.5%) [18].

Machine learning (ML) has become a pivotal tool in predicting the compressive strength of green concrete, which incorporates sustainable materials such as recycled aggregates, fly ash, and blast furnace slag. This approach not only enhances prediction accuracy but also reduces the need for extensive experimental testing, saving time and resources [19]. ANNs are widely used due to their ability to learn from previous data and adapt to new environments. They have shown high performance in predicting compressive strength by analyzing various concrete mix designs [20,21,22]. Techniques like Random Forest (RF), Gradient Boosting (GBM), and Categorical Boosting (CatBoost) have demonstrated superior performance in predicting compressive strength. These methods combine multiple models to improve prediction accuracy and robustness [23,24,25,26]. CatBoost, in particular, has shown high accuracy with R^2^ values of up to 0.979 in training stages and 0.959 in testing stages, outperforming other models [24,25]. SVM and KNN are also effective in predicting compressive strength, with KNN showing higher R values and lower RMSE values compared to other models [27,28]. Hybrid models, such as the combination of Gradient Boosting Regression Trees (GBRTs) with Teaching–Learning-Based Optimization (TLBO), have shown excellent performance with high R^2^ values and low RMSE and MAE scores [26]. Genetic Programming (GP) models have also been proposed for their ability to handle non-linear correlations, achieving high predictive accuracy [29]. The content of cement, fly ash, blast furnace slag, and other aggregates significantly influences the compressive strength. Studies have shown that cement content, fine aggregate content, and water content are critical parameters [26,30]. ML models reduce the need for extensive experimental testing, saving time and resources in the construction industry [20,29]. Advanced ML models provide high prediction accuracy, ensuring reliable and robust concrete mix designs [23,25]. By optimizing the use of recycled and waste materials, ML models contribute to more sustainable construction practices [20,21].

Overall, previous studies highlight both the potential and the challenges of reusing recycled GFRPs in concrete, indicating the need for more systematic and predictive approaches to evaluate their influence on mechanical properties.

## 2. Methods for Reusing GFRP Waste in Concrete

The techniques and methods for reusing and recycling GFRP waste in concrete can be categorized into two main approaches, distinguished by the physical form of the GFRP waste used.

### 2.1. Using GFRP Waste as a Powder or Fine Aggregate Substitute

This method involves mechanically processing GFRP waste (by grinding) into a fine powder. The GFRP powder is used as a partial replacement for fine aggregate (sand) in the concrete mix. In this approach, GFRP powder generally acts as a filler material. The dry constituents (cement, fine aggregate, and coarse aggregate) are mixed. The GFRP powder is added as a substitute for a percentage (e.g., 5% to 50% by weight) of the fine aggregate. It often leads to a reduction in the compressive strength and density of the concrete, but it can be suitable for non-structural applications like lightweight blocks or paving slabs.

### 2.2. Using GFRP Waste as Fibrous Reinforcement

This method focuses on recovering or processing GFRP waste into discrete, slender elements that act as reinforcement within the concrete matrix. This approach has several sub-methods:

(a) Using Recycled Short Fibers

GFRP waste is shredded or milled to produce short, randomly distributed fibers. This method significantly improves flexural (bending) strength and helps control crack propagation in cement sheets and panels.

(b) Using “FRP Needles” or “Slender Elements”

Waste GFRP bars or profiles are cut into short, slender cylinders or needles with a high length-to-diameter ratio. These elements are added to the concrete mix to partially replace coarse aggregate. This enhances energy absorption and toughness under compression and tension, with a slight reduction or minimal change in compressive strength.

(c) Using Macro-Fibers

Decommissioned GFRP components (wind turbine blades) are saw-cut into thin, flat strips with defined dimensions to function as macro-scale fibers. This method provides the most significant enhancement. The macro-fibers are added to the concrete mix at specific volume fractions (e.g., 0.5%, 1.0%, and 1.5%). This greatly improves flexural strength, splitting tensile strength, and toughness, leading to a more ductile failure mode. The concrete exhibits strain-hardening or gradual softening behavior after cracking.

## 3. Research Significance

In the literature review conducted, it was seen that comprehensive artificial intelligence-based research on the parameters affecting the compressive strength of concrete prepared with recycled GFRP materials in different forms, including powder, fibers, and GFRP aggregates, has not been conducted. And this paper is the first, based on previous studies, to investigate, from the perspective of artificial intelligence and machine learning, the behavior of green concrete prepared with recycled GFRP materials as additives, the utilization of which can be beneficial from an environmental perspective as well as for the mechanical strength of concrete. Also, this paper provides a good dataset on the effects of adding recycled GFRP materials to concrete. Using the XGBoost machine learning model, which is an efficient model for fitting numerical and categorical data, a prediction of the mechanical strength of concrete is fitted. Finally, using SHAP and PDP analysis, the effect of all parameters on compressive strength is determined globally and locally.

## 4. Dataset

To investigate the effect of recycled GFRP materials on the compressive strength of concrete, a dataset was collected from reliable sources, which included 119 experimental samples [9,10,13,31,32,33,34,35,36,37,38,39,40,41,42,43,44,45,46,47,48]. This dataset included concrete samples that were subjected to 28-day compressive strength tests, and the compressive strength values were considered as outputs. The input variables of the problem included fine aggregate (FA), coarse aggregate (CA), cement, water, water-to-cement ratio (W/C), amount of recycled GFRPs, and GFRP size. The units of the input parameters of the problem for fine aggregate, coarse aggregate, cement, water, and amount of recycled GFRPs were determined as kg per unit volume of one cubic meter. There were three types of recycled materials in the data, fiber, coarse, and powder materials, which were modeled categorically and assigned to the powdered recycled material type (label 1), the coarse recycled material type (label 2), and the recycled fibers type (label 3) so that the machine learning model could predict the data for the regression problem. The size variable along with this labeling can determine the effect of the recycled shape type of GFRP materials on the compressive strength of concrete.

**Figure 1:**
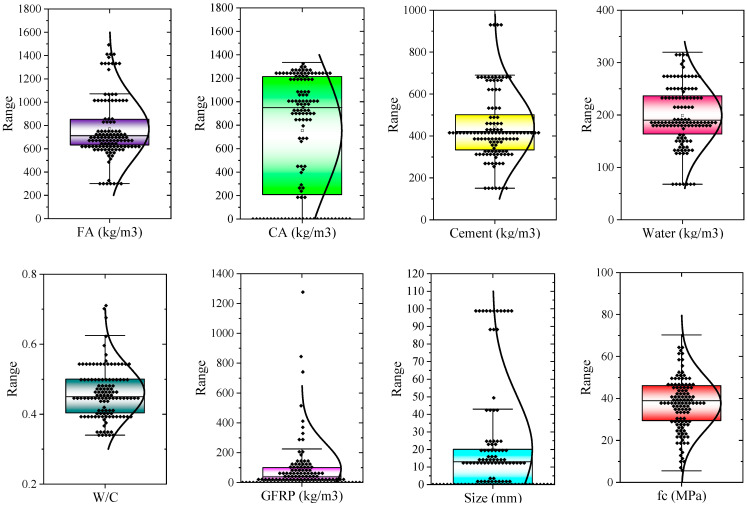
Box plots for parameters of dataset.

In Table 1, statistical characteristics, including minimum, maximum, mean, standard deviation, first quartile (Q1), and third quartile (Q3) values, are shown for all parameters. Also, the number of extracted data (N) is 119, 80% of which was allocated for training the model and 20% for testing the machine learning model. Figure 1 shows the data distribution along with the box-plot curve of the data, the normal distribution corresponding to the data, and the median values of the data. As can be seen, all the data is distributed with a proper density and distribution.

**Figure 2:**
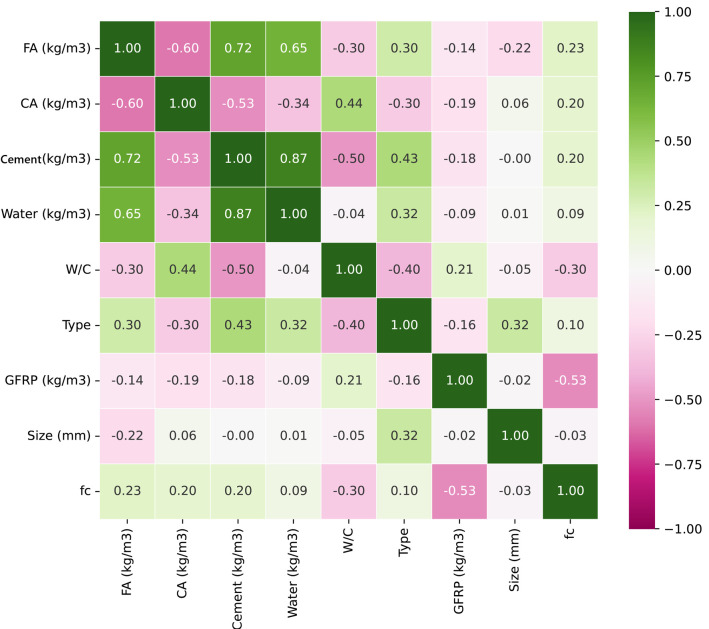
Correlation coefficients for all parameters.

In order to properly understand the relationship between the inputs and the outputs of the problem, a correlation coefficient matrix for the variables was used, which is shown in Figure 2. This plot actually shows the linear correlation coefficients between the inputs and the outputs.

In Figure 2, it can be observed that the parameters of FA, CA, and cement content have a positive correlation with compressive strength between 0.1 and 0.23. The type of GFRP recycling has a positive correlation of 0.1 with compressive strength. Regarding the correlation between water and compressive strength, a negligible correlation coefficient of 0.09 can be seen, which can be considered practically no correlation, but, in contrast, the W/C ratio has a correlation coefficient of −0.3, which shows that there is a negative relationship between this ratio and the compressive strength of concrete. This is an obvious relationship: as this ratio increases, the compressive strength of concrete decreases, independently of the type of concrete. Also, a strong inverse relationship, −0.53, can be seen between the amount of recycled GFRPs and compressive strength, which has been proven in many previous studies. Almost no strong linear relationship can be observed between the variables and the compressive strength of concrete, and it is felt that a more sophisticated or complex model, such as a machine learning model, is needed for regression.

## 5. Machine Learning Model Performance Assessments

### 5.1. XGBoost Model and Optimization

The XGBoost model was utilized to predict the compressive strength of concrete prepared with recycled GFRP materials, which has already been used in many prediction models for various engineering problems [49,50,51,52]. Regression is one of the most powerful and common applications of XGBoost. It extends all the general advantages of XGBoost to predicting continuous numerical values. XGBoost for regression is an ensemble of regression trees. The regression part is defined by the objective function used, which for standard regression is minimizing the squared difference between the prediction and the actual value. Key advantages of XGBoost for regression include its high accuracy and capturing of non-linearity, its robustness to outliers and irrelevant features, and its powerful handling of mixed data types and interpretability (compared to Neural Networks). In these models, there are many hyper-parameters whose values must be selected appropriately so that the predicted response is close to the actual value. Given that it is difficult to determine these parameters in a normal way, optimization methods such as the Bayesian optimization (BO) method, which is one of the hyper-parameter optimization methods, should be used. The hyper-parameters of this model are divided into four categories: General, Booster, Learning Task, and Command Line, and each of these categories has other subcategories. In this paper, the hyper-parameters, colsample by tree, learning rate, maximum depth, number of estimators, subsample, and random state were selected for optimization. After the optimization process using the Bayesian method, the optimal value for each parameter is shown in Table 2.

### 5.2. Model Performance Evaluation

**Figure 3:**
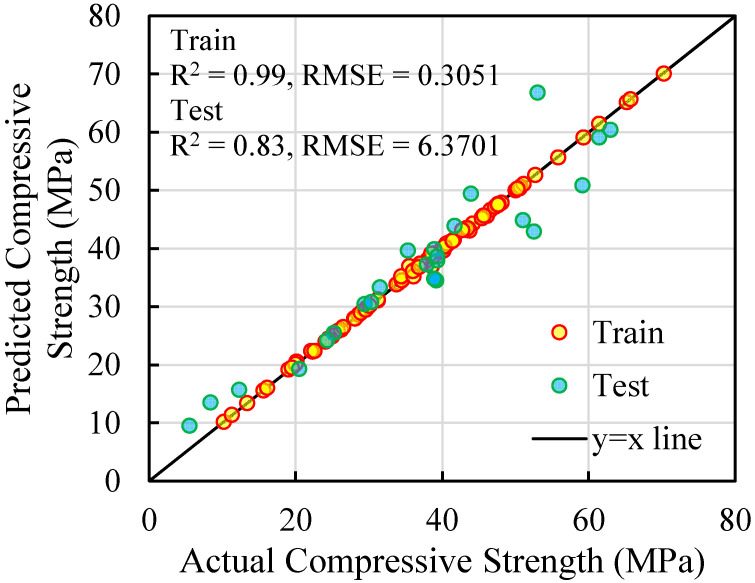
Actual and predicted values for compressive strength (CS).

According to the XGBoost model whose parameters were optimized, 80% of the data was allocated for training and 20% for testing the model. Figure 3 shows the actual compressive strength values and the predicted compressive strength values generated by the XGBoost model. As can be seen, there is good agreement between the predicted values and the actual values.

To evaluate the quality of model fitness, various indices can be used. In this paper, the coefficient of determination (*R*^2^), which indicates the proportion of variance in the experimental data explained by each model; the root mean square error (*RMSE*), measuring the average magnitude of prediction errors; the mean square error (*MSE*); and the mean absolute error (*MAE*) were used [49]. These indices are used in many studies. The mathematical formulae of these indices can be seen in Equations (1)–(4).

(1) $$R^{2}=\frac{{\left(\sum_{i=1}^{n}\left(CS_{ac}-{\bar{CS}}_{pr})(CS_{pr}-{\bar{CS}}_{pr}\right)\right)}^{2}}{\sum_{i=1}^{n}{\left(CS_{ac}-{\bar{CS}}_{ac}\right)}^{2}\;\sum_{i=1}^{n}{\left(CS_{pr}-{\bar{CS}}_{pr}\right)}^{2}}$$

(2) $$MSE=\frac{\sum_{i=1}^{n}{\left(CS_{ac}-CS_{pr}\right)}^{2}}{n}$$

(3) $$RMSE=\sqrt{\frac{\sum_{i=1}^{n}{\left(CS_{ac}-CS_{pr}\right)}^{2}}{n}}$$

(4) $$MAE=\frac{\sum_{i=1}^{n}\left|CS_{ac}-CS_{pr}\right|}{n}$$

where $CS_{ac}$ is the actual compressive strength of concrete prepared with recycled GFRP fibers, $CS_{pr}$ is the predicted compressive strength from the XGBoost model, and n is the number of samples used in the training and testing phases. These values were calculated for both the training and testing phases and are shown in Table 3. It can be seen that the R^2^ value for both the testing and training stages is above 0.8, which is an acceptable value. The error value in the testing stage of the model is slightly high, but considering that the data has three different general categories, fiber, powder, and coarse grains, it has provided acceptable values for predicting the data. In fact, this model can be used for any type of recycled material shape, which makes it a practical model.

**Figure 4:**
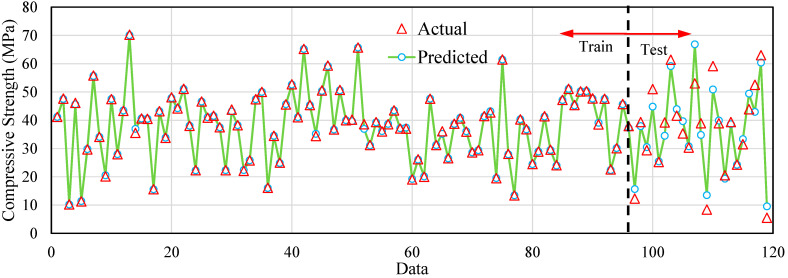
Actual and predicted values for data samples.

In Figure 4, the predicted values are shown with lines and circular dots, and the actual values are shown with triangular dots. It can be seen that a very good match between the data and the model is recorded in the training data section. Given that the data is classified into three categories based on the type of recycled GFRP material, the prediction of the data is based on the size of the material as well as the type of material. For this reason, it is often difficult to provide a model with high accuracy for such data that has categorical variables. For this reason, it can be observed that the error increased to a certain extent in the testing phase. However, the data match and also the prediction process seem reasonable.

### 5.3. Feature Importance

**Figure 5:**
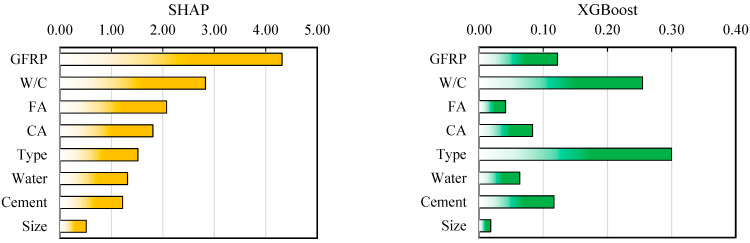
Feature importance for XGBoost and SHAP analysis.

To determine which parameter has the most influence on the output, various tests can be used. The XGBoost model itself has a built-in feature importance determiner that can be used for decision making. Also, SHAP analysis can be used to determine feature importance, and it is used in many machine learning analyses [53,54,55,56,57]. Figure 5 shows the score for or importance of each variable in the XGBoost and SHAP models.

In the XGBoost model, it can be seen that the parameters of the type of recycled materials and the W/C ratio are the most important and that, after them, the amount of cement, the amount of GFRPs, CA, water, FA, and the size of the recycled materials are of great importance. It seems that, according to the results, this importance is not very reliable, and for this reason, the SHAP model was examined. In the SHAP feature importance model, the amount of GFRPs and the W/C ratio are more important. After these, the characteristics of the aggregate materials, including CA and FA, are of great importance. The type of GFRP recycled materials is also of great importance. In both models, the size of the GFRP recycled materials is of less importance. The importance of the data types and their scoring values are such that no variable can be excluded from the analysis, and therefore all variables are considered in the analysis. XGBoost may bias feature importance due to the specific nature of the model and the way in which SHAP values can inflate feature importance scores due to model biases. The lack of real values complicates the validation of feature importance, as biased feature importance can obscure the true relationships with the target variables. However, this feature importance problem can be largely controlled based on engineering judgment and some statistical analysis. However, in this section, since the SHAP method was used for feature importance, the rest of the steps are presented based on this method [58].

**Figure 6:**
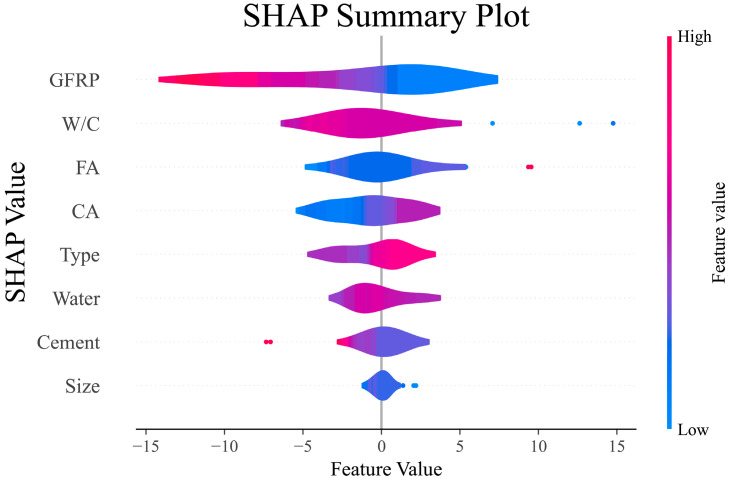
SHAP violin plot for features.

Figure 6 shows the violin curve for the variables based on the SHAP analysis. According to this curve, the range of the parameters’ influence, as well as the positive or negative effects of the variables on the responses, can be observed. Unlike Figure 5, where only the importance value of each variable can be observed, in this curve, the magnitude of the effect and the direction of the effect can be identified.

Based on Figure 6, it can be seen that the amount of GFRP recycled material has a very large effect on the compressive strength. The data skewing towards the negative side of the curve indicates that the amount of recycled material may have a negative effect on the compressive strength response. Further analyses performed with the model are needed to determine the effect of this parameter. Also, the effect of the W/C ratio on the compressive strength of concrete is negative. As the water-to-cement ratio in concrete increases, the compressive strength of concrete decreases regardless of the type of fiber or plain concrete. The effect of other parameters was investigated based on PDP analysis. Considering that the type of GFRP recycled material has a positive role in this type of analysis, it should be investigated which types of recycled material, powdered, fibrous, or plain grain material, have a positive effect and which types of material have a negative effect.

### 5.4. Partial Dependence Plots (PDPs)

**Figure 7:**
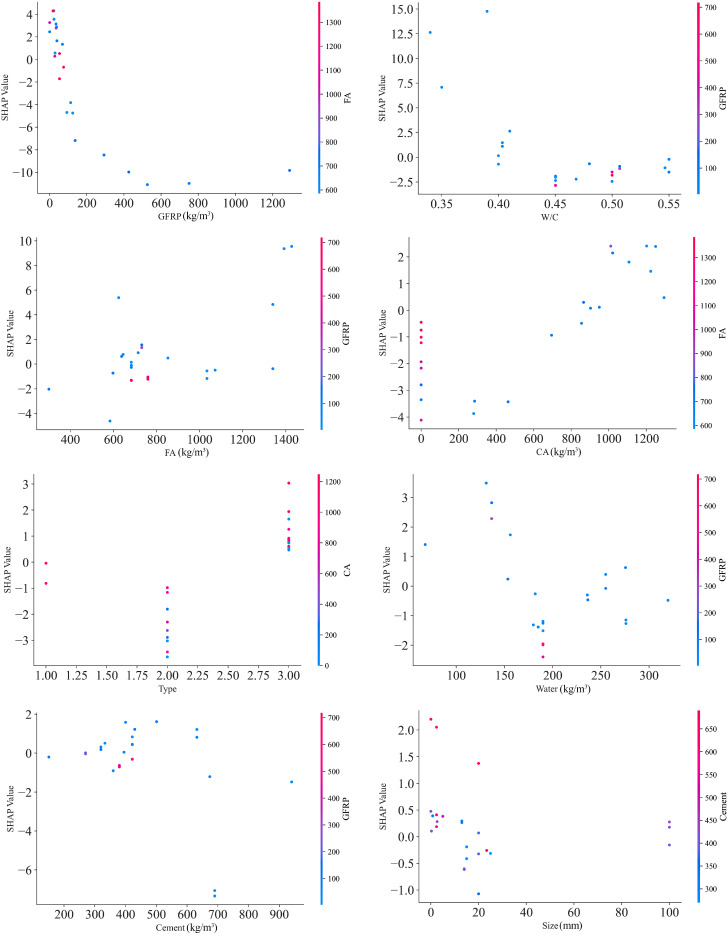
SHAP PDP plots for features.

Information on the interaction of variables and the trends of variables is required, so PDP analysis was selected. Figure 7 shows the results of the PDP analysis for all variables and the compressive strength of concrete. According to the figure, it can be seen that increasing the GFRP content shows a generally negative trend regarding fc in this dataset. Positive or neutral effects can be observed mainly at low dosages. The apparent threshold near ~100 kg/m^3^ should be considered dataset-specific and warrants validation. That is, adding GFRP recycled materials beyond a certain limit reduces the compressive strength of concrete. This behavior can also be seen in other fiber concretes, which, in addition to reducing the efficiency of concrete, plays a role in the load-bearing matrix and the coarse-grained performance. This can be the subject of a new study on the application range of GFRP recycled materials and their types. It can also be seen that the W/C ratio also has an inverse relationship with the compressive strength, which is representative of SHAP. Up to a ratio of about 0.4, it can have a positive effect, but values greater than that have negative effects.

The effects of FA and CA are often seen as positive. But these effects are greater for coarse grains. A direct relationship between coarse grains and SHAP values was observed, which can also be seen in other concretes. In examining the type of recycled materials, it was seen that type 1, the powdered type, has a relatively positive effect on compressive strength and that it can have a positive effect on the environment and mechanical properties of concrete in laboratory models. The second type, GFRP coarse grains, has a negative effect on resistance because these materials have high resistance to coarse-grained aggregates and for the same reason can weaken the compressive strength. In comparing aggregates made from GFRP recycled materials and stone aggregates, it was seen that the stone aggregates had a higher resistance. However, the powder form of these materials, which can play the role of filler, is noticeable in the process of increasing the compressive strength of concrete. Also, the fiber type (label 3) also had a significant effect in terms of increasing resistance.

### 5.5. Local SHAP Analysis

In order to examine the behavior of the data locally, two laboratory samples were selected [38,48] and SHAP analysis was performed on them to examine the accuracy of the model and determine the effect of each parameter in terms of reducing or increasing the compressive strength. The mixing design for these two samples is given in Table 4.

**Figure 8:**
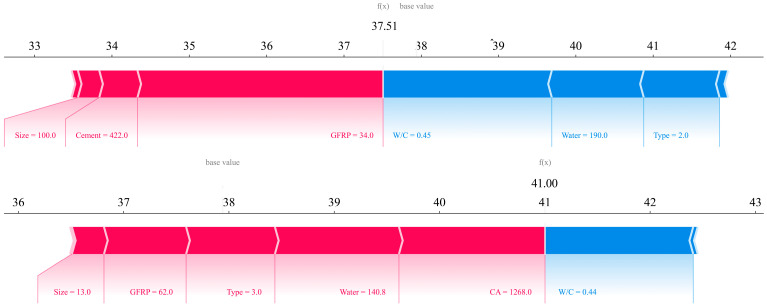
Force plots for sample 1 and 2 based on SHAP.

In Figure 8, the force-plot curve for these two samples is shown. It can be seen that the compressive strength value predicted by the XGBoost model for sample number 1 is 37.51 MPa and that the compressive strength value for sample number 2 is 41 MPa. The accuracy of the model is evident in these two samples. The compressive strength values given in Table 4 are actual values, and the values given in the force plot are predicted values.

In the study of sample 1, it can be seen that the parameter of the type of recycled material, which is recycled GFRP aggregate (label 2), causes a decrease in strength. Also, the parameters of water and W/C ratio cause a decrease in strength, and the parameter of the amount of GFRPs, which has the greatest effect, causes an increase in strength. Also, the amount of cement and the size of the fibers are also effective in increasing the strength. In sample number 2, only the high W/C ratio causes a decrease in strength. And the parameters of the type of recycled material, which is fiber (label 3); the amount of coarse grains; the amount of water; and the amount of GFRPs and their size cause an increase in strength. The study of the type of increasing and decreasing parameters was logically performed on two laboratory samples, and it was seen that in addition to the exact value of the compressive strength, a logical effect on the parameters can also be observed.

## 6. Limitations and Future Studies

The data used in this article is based on the latest articles available in this field, and it seems that more data from the laboratory will help to better understand the behavior of this type of concrete. Also, there is less laboratory data for concrete prepared with GFRP powder than for the other sample types. In this article, only the XGBoost machine learning model was used. Statistical data mining methods can also be used to investigate this issue. In the field of investigating the effects of mixing concrete with recycled FRP materials in designs optimized by artificial intelligence, there has been no comprehensive research so far, but it seems that an optimal mixing design can be achieved by using the results of artificial intelligence analyses and the recursive design method.

## 7. Conclusions

This work used machine learning to predict the strength of concrete made with recycled fiberglass (GFRP) waste. It found that keeping the GFRP amount under 100 kg/m^3^ and using it as a powder or fiber, rather than coarse chunks, leads to stronger concrete. The model provided a reliable guide for using this waste to create more sustainable construction materials. Based on the results, the following concluding remarks are made:

- The incorporation of recycled GFRPs was found to be most effective at a limited dosage. Compressive strength was observed to decrease significantly when the GFRP content exceeded the threshold of 100 kg/m^3^.
- A strong negative correlation (−0.53) was quantified between the amount of GFRPs and the compressive strength, confirming its detrimental impact at higher volumes.
- The form of the recycled material was identified as a critical performance factor. Powder (type 1) and fibrous (type 3) forms were shown to have a positive effect on strength, while the coarse aggregate form (type 2) was consistently associated with a reduction in strength.
- The machine learning model, XGBoost, was developed and achieved a high predictive accuracy, evidenced by a coefficient of determination (R^2^) of 0.8284 and a root mean square error (RMSE) of 4.37 MPa on the test dataset.
- Through SHAP analysis, the water-to-cement ratio (W/C) and the GFRP amount were quantitatively confirmed as the two most influential input parameters on the model’s output, governing the compressive strength.
- Based on partial dependence plots (PDPs), the water-to-cement ratio was shown to have a negative relationship with strength, with optimal performance observed at a ratio below 0.4.

## Figures and Tables

**Table 1:** Descriptive statistics for dataset.

Parameter	Minimum	Maximum	Mean	Standard Deviation	(Q1)	(Q3)
FA (kg/m^3^)	300.4	1503	770.61	265.93	632	852.8
CA (kg/m^3^)	0	1335	753.63	497.5	207.5	1215
Cement (kg/m^3^)	150.8	940	442.57	165.01	333	501
Water (kg/m^3^)	67.9	319.6	199.097	58.43	163.4	236.5
W/C	0.34	0.71	0.46	0.072	0.40	0.5
GFRPs (kg/m^3^)	0	1292	92.02	170.83	14	99
Size (mm)	0	100	20.17	30.50	0.25	20
Type	Powder = 1		Coarse aggregate = 2		Fiber = 3	
f_c_ (MPa)	5.5	70.25	37.72	12.84	29.4	46.11
N	119					

**Table 2:** Optimized values for hyper-parameters of XGBoost model.

Hyper-Parameter	Colsample by Tree	Learning Rate	Maximum Depth	Number of Estimators	Subsample	Random State
Optimum value	0.9155	0.1081	8	87	0.9746	42

**Table 3:** Statistical indices for model evaluation.

Phase	*R* ^2^	*MSE*	*RMSE*	*MAE*
Train	0.9994	0.0931	0.3051	0.1492
Test	0.8284	15.31	6.3701	2.6965

**Table 4:** Experimental samples for local SHAP analysis.

Parameter	FA (kg/m^3^)	CA (kg/m^3^)	Cement (kg/m^3^)	Water (kg/m^3^)	W/C	Type	GFRPs (kg/m^3^)	Size (mm)	f_c_ (MPa)
Sample 1 [38]	683	903	422	190	0.45024	2	34	100	37.9
Sample 2 [48]	632	1268	320	140.8	0.44	3	62	13	40.9

## Data Availability

The original contributions presented in this study are included in the article. Further inquiries can be directed to the corresponding author.
